# An Address on Obstetric Medicine

**Published:** 1873

**Authors:** J. Braxton Hicks

**Affiliations:** Obstetric Physician to Guy’s Hospital; President of the Section


### AN ADDRESS PREPARED FOR THE OPENING OF THE SECTION OF OBSTETRIC MEDICINE.

*At the Annual Meeting of the British Medical Association in London, August* 6, 1873.

By J. Braxton Hicks, M.D., F.R.S.*

*Obstetric Physician to Guy's Hospital; President of the Section.*

*Gentlemen,*—When the honor of the Presidency of this Section was offered me, I felt, as I have no doubt my predecessors had done, a difficulty in selecting a subject with which to introduce, as is expected of the President, the business of the meetings.

Taking into account what had already been given, and thinking the papers to be read would have mainly a practical tendency, it occurred to me that a few remarks on the more recent advances, both in the anatomy and the physiology of the parts connected with our department, would not be out of place at this time.

I am quite aware that with the majority, especially of those actively engaged in practice and the stern necessities it demands, there is a yearning after that which appears more immediately practical; yet who can say what the bearing upon practice of the revelation of any new facts shall have? Who can define at once their relations to the practical wants of mankind?

Gentlemen, whatever advantages there may be in the division of our profession into sections, it will be of great disadvantage if each branch fail to employ fully the opportunities such a subdivision of labor gives to it, and a still greater disadvantage if it fail to make use of the information which the workers in other departments have by their strenuous assiduity gained. By this very subdivision knowledge rapidly advances, the discovery of a new principle changes the whole of practice, and unless constant intercommunication is made, a section in a year or two may be left far behind. But I am sure you appreciate all this, and will agree with me that unless we are guided at every step of practice by scientific accurate investigations, we can never be sure we are in the right path, nor feel confident we may not have to retrace our steps.

The short time allotted to me does not permit me to do more than allude to a few points, and I must ask your indulgence on this account, though were it longer, I might have to crave it for the opposite reason.

There are many aspects in which we may view the sexual organs of women, each one presenting an appearance more or less distinct from the other. Thus we have the parts concerned in the development and discharge of the ovum; then the organs concerned in impregnation, generally, but not necessarily mixed with sexual desire; and again, there is the wonderful growth of the muscular element, and consequently the enormous increase of expulsive force, together with the laws which govern the mechanism of that force, and its effects. The first—namely, the formation of the ovum—is connected with its cell elements; the second involves largely the nervous element, both sympathetic and spinal; the third involves the nervous element acting upon the organic muscular fibres, and developed, the one certainly, both probably, beyond all comparison with any other parts of the body. The effects of the force bear rather upon well known laws of mechanics, for they depend upon power and opposition; and, as a great obstetrician remarked, given the physical condition of the passage and of the fœtal head, one could beforehand pronounce the exact motion. It would be flattering ourselves if we asserted that our knowledge is perfect in either of these directions. In common with our colleagues in other departments, we must confess much uncertainty in our knowledge of the intimate structure of various elements, of the physiology of many parts, and of the forces developed by the action of the uterus. Still there is no reason to despair of some day possessing a complete knowledge, and very much has been done of late years. I would take this opportunity of pointing out to the more ambitious of my younger colleagues, how excellent an opportunity is afforded them of laying the foundation of a solid reputation.

Having said this much, let us proceed to pass in review the more recent advances of importance.

Within the last few years the opinions as to the development of the ova and graafian follicles have undergone much change. So long ago as 1859, E. Pflüger published in a paper, *Uber die Bewegungen der Ovarien*, in Reichart and Du Bois-Reymond's *Archiv*, an opinion that the Graafian follicle originated in tubes, and he pointed out therein the analogy to the seminal tubes of the testis, and that these were formed in the stroma, and continued to be so even after the birth of the fetus. In near accordance with this view is Kolliker's; and more recently Waldeyer, in a work, *Eirstock and Ei* (1870), and still later, in an article in Stricker's "Histology," while agreeing as to the tubular origin of the Graafian follicles, has pointed out that these tubes are formed by the inflection of the surface of the ovary around; that the ovary is covered by columnar epithelium, as is the case in serous membranes; and that this epithelium lines the inflection throughout. He then points out that among the epithelium from very early life (four days in the chick), there are enlarged epithelium cells, and these are drawn into the inflected portion, and ultimately become the ova. He says that the ordinary epithelium cells about these larger cells arrange themselves so as to surround them, and form not the Graafian follicle, but the outer membrane of the ovum or zona pellucida. Where there are many of the large cells, the tubes separate up into as many ova, and these become so many centres of Graafian follicles. Waldeyer further says, that in all animals the ova seem to be simply more highly developed epithelial cells of the ovary, which have undergone some peculiar modification, so that the follicular epithelium and the egg-cells stand in direct generic relation to each other. Waldeyer's account is generally well received, though hardly sufficient time has elapsed to give confirmation or refutation to his opinion.

One is tempted here to ask whether these cells have any more direct and immediate descent from the photoplasmic matter of the ovum, which has only itself just started into life. It is only a few hours since it assumed activity, and at once it multiplies certain cells, which it stores up, and which endure without renovation till required for reproduction. Are these immediate descendants of the mother ovum, or are they, like the other cells of the body, differentiated off by the general laws which cause this also wonderful phenomenon? One is tempted also to remark upon the force which is evoked by the fusion of the male and female elements. It may ultimately prove to be simply chemical; in any case, it is the highest example of what we may still, for the want of a better name, call vital force. And it has this peculiar property, that the product of the fusion not only holds the force within itself, but upon the tissues upon which it rests it acts as a powerful stimulant, and that not tending to its destruction, but enduring it with increased vitality, whereby each elementary tissue is increased and multiplied.

Simultaneously with investigations on the nerve distribution in other parts of the body, attention has been directed to their arrangement in the uterus and the vagina by various observers. The investigation of the termination of the nerves in the non-striated muscular fibre of the uterus will be acknowledged by all to be a very difficult matter. Still the researches of Frankenhäuser, in his work " On the Nerves of the Uterus, and their Mode of Termination in the Smooth Muscular Fibres" (1867), agree very much with the researches of J. Arnold in his articles on "Organic Muscles" in Stricker's Histology. They have, at least, traced the ultimate fibre into the interior of the nucleus of the muscle-cell, but they differ as to whether the extremities end by a plexus in the nucleus, or end in its granules. In any case, so close and intimate a relationship exists between nerve and muscle, that any one can hardly look upon muscle as otherwise than the outward expression or exponent of nerve force.

The termination of nerves on the inner surface of the uterus has not yet been satisfactorily traced out. A nerve has been said to pass into each papilla of the cervix, and still further, Lindgren even finds a fine plexus of granular masses, which, breaking up in a brushlike manner, extend to the epithelium. Upon this point, however, there is some doubt, though it is much in accord with what has been noticed in other surfaces.

Very recently the termination of the nerves of the vagina has been made the subject of a paper by Chrschtschonovitch before the Imperial Academy of Sciences of Vienna. Here it is stated that there is a network beneath the epithelium from which branches pass between the epithelium cells, but which do not join the connective tissue cells. This arrangement much approaches the description given by Klein, in *The Quarterly Journal of Microscopic Science*, of the peripheral distribution of non-medullated nerves on the cornea, for he says that the fibres, having reached the base of the epithelial cells on the anterior surface of the cornea, and forming there a deep intra-epithelial network, run up between the cells and form plexuses between the superficial layers, being all but exposed on their surfaces, from which, indeed, they are separated by only one or two of the laminated cells, and form here a superficial intra-epithelial plexus. This arrangement is not peculiar to these mucous membranes at least, the close contact of the epithelium with the ends of the nerves is marked in all—so much so, that in some the nerves appear to enter the epithelial cell. Besides this, the recent investigation of Frankenhäuser, Koch, Kehrer, and others, show that, as in other parts of the internal organs, there are ganglia in the submucous membrane with which two or three pale nerve fibres are in connexion. These researches point out to us that we can not ignore in our clinical pathology—indeed, that we must give a prominent place to—the nerve-element. May I be permitted to make the remark that this very free inblending of the nerves with the other tissues has not received—at least, judging from the writings of authors—that general consideration which it deserves? Blood-vessels and exudations, mechanical pressure and obstructions are freely mentioned; and instruments are used as if the uterus were a simple elastic tissue to be dilated by main force, like a piece of india-rubber. Considering the large nervous supply to the os and cervix uteri, we can understand the great effect, of a reflex character, which a small abrasian may produce; and can point out a satisfactory explanation to those who still remain incredulous to the practical experience of all gynecologists. Obstetricians, more than others, have the opportunity of viewing the effect of the reflex function, both of the spinal and sympathetic systems. The uterus is almost the only organ which, endued in the main with nerves from the sympathetic, is capable of being seen and freely handled. It should neither cause surprise nor disbelief if the phenomena recorded by careful observers do not in degree, or exactly in kind, tally with observations made upon organs not so much dependent on the sympathetic nerves. Then, again, the addition of sexual desire can never be completely left out of consideration, and often has to receive considerable attention, both in explanation of the phenomena which are brought before us and in the correction of the abnormalities.

Since the admission that the ovaries are a very influential factor in many of the great phenomena of woman's life, we can not, in any case, leave their influence out of consideration, but this only removes the question one step back, and it still remains to be explained what influence it is which excites the ovaries, and how it is set agoing. It may be the regular growth of the ovum in the Graafiian follicle which stimulates the nerves by pressure, causing an increased flow of blood and some congestion, and this by putting pressure on the contents of the ovary causes the ripe follicles to burst. The same irritation causes stasis in the blood-vessels of the uterus, a rapid growth of the mucous membrane elements, and the other phenomena of menstruation ensue. Let any one listen carefully to those who suffer at the menstrual period, and it will readily be perceived how widely the general nervous system is affected by the nerve disturbance of the uterus. Need we then wonder at the distress which, in highly sensitive conditions of the system, is felt in the various other disorders of the uterus?

Neither is there any other organ of the body which grows as the uterus does in pregnancy. This is, of course, through the agency of nervous stimulation and not from immediate contact; because the uterus grows in a marked manner in extra-uterine pregnancy where the ovum is some distance from the corpus uteri. And this brings me to the question, whether the once disputed point, as to whether the nerves of the uterus grew during pregnancy, has received any recent answer. Dr. Chrobak, in his article on the Structure of the Uterus, in Stricker's "Histology," makes this remark: "There is an indubitable growth of the nerves also during pregnancy, (Tiedemann, Remak, and others,) and, according to Kilian, the double-contoured nerves may be followed further in pregnancy than in the virgin state." If decentralization of the sympathetic nerve be the character of its arrangement, then it is easy to understand that, although an increase corresponding to the growth of the muscular tissue may take place, yet this dces not necessitate a large increase of the nerve-trunks which come from the hypogastric plexus.

I feel that I have already detained you long enough on anatomical points, but must just mention some interesting observations made by Kundrat (*Medizin Jahr*, vol. ii., 1873, and *Med. Times and Gazette*, July 26th, 1873,) on the mucous membrane of the uterus before, during, and after menstruation. He describes the ordinary characters of the mucous surface as markedly altered at the monthly period of the uterine activity; it is swollen, thick, and loose, and almost diffluent, covered with a whitish or bloody mucus, freely injected in parts, and in many cases uniformly colored of a deep red. A microscopical examination reveals increased abundance of the cellular matrix, with great elongation and dilatation of the glands. But what he especially points out is, that the condition of the uterus just described probably precedes the occurrence of the discharge of the ovum, and what is perhaps more striking the menstrual flow by several days. The uterus appears prepared for the receipt of the ovum a certain time before the rupture of the Graafian vessicle. At the menstrual flow, and for a short time after it has ceased, careful examination shows a remarkable change in the microscopic appearances. The cells of the stroma and the vessels, as well as of the epithelium of the glands and surface, are dull in appearance, and filled with fat granules. Kundrat believes that the hemorrhage does not cause the fatty change, but is caused by it. The anatomical sequence of events is the swelling of the mucosa, fatty change in the cells and vessels, vascular rupture and hemorrhage. The type of the impregnated uterus is seen in the active uterus when the mucosa is swollen and menstruation has not yet commenced. If the bleeding does commence, it is a sign that the ovum has perished, and that the mucosa is returning to its state of rest. According to this view, a developing ovum or growing embryo belongs, not to a menstrual period just past, but to one just prevented by fecundation.

One of the most important questions in obstetric physiology under discussion of late, has been that of the forces employed in labor. Many have raised the question, as, for instance, Haller, and Lithopædus Scriverensis, quoted by Swift in his "Tale of a Tub." Both these placed the force at a high point— the latter, indeed, at 400 pounds. The question, however, remained latterly in abeyance until Dr. Matthews Duncan, and, at the same time and independently, Poppel, of Munich, carried out numerous experiments on the power required to burst the membranes. Dr. Duncan found the toughest membrane of an easy labor to require twenty-nine pounds for an os uteri of four and a half inches; Poppel, nineteen pounds for an os uteri of four inches. Two or three years after these experiments, Professor Haughton calculated that the full force of the uterus itself was about thirty-two pounds on an os uteri of four and a half inches, calculating from the area of the uterus, the thickness of its walls, and its curvature. Having obtained this result, he argues that, as nature does not employ more power than is required, the resistance of labor is only somewhat below this. But, in calculating the force of the accessory powers, he came to the conclusion that they were far in excess of that of the uterus, equal, indeed, to five hundred and twenty-three pounds on the area of four inches and a half diameter; that is, for the maximum power exerted. It would be out of place to go into the calculations, but I may say that he took two bases of calculation; one the weight of a mass of given size, capable of being lifted on the centre abdomen, and then calculating for the whole area; and, the other, the thickness, area, and curvature of the abdominal muscles. He assumes that the uterus itself does no more than rupture the membranes, and that the auxiliary forces do the major portion of the remainder.

It is evident that, before this important point is thoroughly cleared up, some further experiments must be made; because

I think, most persons accustomed to operative midwifery would find it difficult to acknowledge that a force equal to a quarter of a ton can be, in even the most forcible labor, put on the parturient passage. Either some loss of force takes place, or else Professor Haughton has overstated the strength of the muscular tissue of the uterus and the auxiliary forces. No doubt, where the waters have escaped, the pressure is not so direct nor so great as when plenty of fluid is present, because a considerable amount of force is exerted laterally against the body of the child; and we know that, in some forms of labor, although the uterus is acting most violently, the effect on the fetal head, *quoad* descent, is next to nothing, the whole force being exhausted in compressing the child, to its great detriment. Again, although in easy labors the contractions of the body and fundus take place universally on the contents, yet those who believe in the peristaltic nature of the contraction will see that the whole of the uterus is not engaged in the action at any time, and that something must be taken off its total force when considering its expulsive power.

The power of the uterus could be roughly reckoned by finding the tensile resistance of its attachments. Now we know that in some cases the uterus does rend its attachment to the vagina: in ordinary cases, of course, it does not, but we may say roughly that the force of the uterus alone is rather less than the total resistance of its attachments—*i. e.*, the vagina, the round ligament, and the peritoneal reflexions, the broad ligament the principal. Of course the auxiliary forces, by pressing on the abdominal contents, support the uterus, and prevent it from recoiling when the head has escaped, and thereby rending its attachment. But should the auxiliary forces not be capable of acting, as from paralysis, extremely lax abdominal walls, heart or lung mischief, then the force of the uterus, supposing the head has escaped and the uterus meets with an obstacle, is thrown on the attachments. Thus a gauge may be approximately made as to its force. With regard to the auxiliary forces, put at so high a figure by Professor Haughton, I think that his data as to women must be much reduced. The muscles of women are much less and less powerful than those of men; they are seldom in anything like condition. This last observation, however, does not always hold; because in some women who work as hard as men, the labors are very severe, and doubtless in them the total labor power is at the maximum. Supposing Professor Haughton correct in his data, then I think it has yet to be shown experimentally that on the brim or cavity the downward force is so great as he believes, or nearly so.

Some observations have been recently made on the contractions of the uterus during pregnancy. Uterine contractions have been generally recognized toward the end of pregnancy, but in a recent paper I have shown that they occur spontaneously throughout pregnancy, from at least the third month, and are of great value in the diagnosis of pregnancy. The cause has been attracting some attention abroad. Drs. Aser and Schlesinger, in the fifty-second number of the *Contralblatt für die Medizinischen Wissenschaften*, (1871,) pointed out that in rabbits, whenever the blood in the brain or the uterus became highly charged with carbonic acid, tetanic contractions of the uterus occurred. They experimented in various ways with the same result. Excitation by galvanism of nerves, such as the femoral or radial nerve, produced contractions; but when the spinal column was divided as high up as possible, compatible with respiration, the effect was the same, even though the nerve chosen was above the division. The channel by which the effect was carried would seem to have been the sympathetic branches. At any rate, it would appear that the uterus is excited in many ways. But the suggestion which I threw out in the paper to which I have referred, to the effect that the uterine contractions act as a supplementary heart to relieve the stagnant state of the blood in the uterus, receives some confirmation by the first named experiments. Dr. Reimann, of Kiew, has also published some observations on the innervation of the uterus and on its contractions. He experimented on bitches and cats, and subjected the uterus, partly or wholly separated from the body, to various kinds of irritation. He found that the uterus, separated from the cerebro-spinal axis, and even removed from the body, responded to the irritation by peristaltic and rythmical movements of the whole organ, even when only a portion of it had been subjected to the irritation. He also found that the uterus separated from the body, but maintained at its normal temperature, spontaneously contracted and relaxed for about an hour after the death of the animal. Dr. Reimann concludes, from these experiments, that the contractions of the uterus are under the influence of certain organs (ganglionic) not yet anatomically demonstrated, but which are situated in the uterus itself; and that like the contractions of the heart, they are independent of the cerebro-spinal system, though physiological and pathological facts prove that the latter has certain influence over them.

Regarding the great question as to the cause or causes which conduce to set up labor at full term, we are still without definite information. There may be, as indeed I believe there is, more than one factor. But in regard to the direction in which the forces act when once established, there is yet some uncertainty. Does the fetus, during a pain, enter the area of the brim with its long axis coincident with the axis of the brim? If we refer to the description of authors we find them varying very much. Formerly, as most of you remember, it was believed that the axis of the fetus inclined more forward by a few degrees than that of the brim. Some later writers, amongst whom I may name Dr. Leishman, have endeavored to show that they are coincident, or, as it has been called, "synclitic." Again, Schatz and Schultze have been over the subject, and they, in contra-distinction, make out that the axis of the fetus is more perpendicular than that of the brim. This would tend to throw the axis of the head of the fetus against the pubes. The condition of the abdominal parieties would probably regulate the direction to a great extent. Unquestionably the uterus, having fallen forward owing to the relaxed abdominal parietes, would not, even in the most forcible contraction, straighten or erect itself completely. This I have noticed to be the case even when the uterus has not been fully developed.

It has been thought that a lateral inclination of the axis of the uterus has been discovered, and Dr. Matthews Duncan in this country, having followed out this point, has noticed that there is a distinct inclination in the majority of those cases which he has observed. What influence this may have on the direction of the head and upon the relative frequency of one parietal bone overlapping the other, has not yet been determined. More numerous investigations are yet required to establish the value of these observations.

The line of the auxiliary forces is said by Schultze and Schatz to be directed still more in the line of a perpendicular than is the uterine action, so that there is a loss of power by the head being driven against the pubes instead of against the sacrum. But, as Dr. Duncan points out, this condition has been looked upon not as the natural position, but rather as an unnatural one.

Although accuracy on these points may be of little importance in a natural case, because any slight error is readily overcome, yet in difficult cases it is not so. The position of the transverse rupture of the upper vagina and cervix uteri depends on the inclination of the forces. If the fetal head presses with undue angularity, then it can readily be seen how the wall against which the head projects would be liable to rent, provided the pressure be great or the tissues weak.

It is the generally received opinion that the vertical axis of the head in its passage through the pelvis does not correspond with the mesial axis of the pelvis, but latterly Dr. Küneke has, in a work called "The Four Factors of Labor," endeavored to show that it is synclitic throughout: in other words, that not the angle of the parietal bone, but some point in the antero-posterior diameter is the presenting part. This has been contested by Dr. Matthews Duncan, and he asserts that the head descends to the middle of the cavity as it enters the brim, synclitic, because it meets with no opposition, but that after this point the axes never correspond. He and Schrœder observe that the pressure of the head is mainly against the posterior wall of the cavity, and at the point of contact there is retardation by friction of that side of the head to a greater extent than the anterior, which bears but slightly on the pubic bone. A familiar illustration of this mechanical point may be seen on any railway curve. Of course it will occur to many that the variability of the size and shape, both of the fetal head and also of the pelvis, must render this point a difficult one to investigate, for it will always be difficult in any given case to say how far the conditions of both were normal. It can only be by observation of a large number of cases that the natural tendency can be made out.

The examination of the form of the head after delivery, and particularly after laborious labor, is a point which has perhaps not received the attention which it deserves. Many practical hints might be gathered in this way.

Tied as I am to time, I must omit much interesting matter regarding the circumstances which determine the various presentations and positions of the fetus. Of late years, as most of you are aware, much information has been gained on these points. It was also my intention to have spoken a little in detail of a subject of great interest to all engaged in obstetrics and gynecology. I refer to what Dr. Matthews Duncan has called *the retentive power of the abdomen.*

But I must hasten to point out that Schultze and Duncan have taken up a very interesting but surprisingly neglected subject—namely, the mode by which the placenta is naturally expelled. Baudelocque and Schultze agree in saying that blood is effused between the placenta and the uterus, and thus, having been dislodged nearly to the os uteri, the placenta is expelled by a contraction. Duncan asserts that this mode is not to be considered natural, but that the placenta is, in a normal state, rolled together in a long roll, with its amnial surface inside; this shape adapts it to pass readily through the os uteri. I believe further observations will confirm Duncan's assertions, so far as those cases where the placenta is attached to the side of the uterus more than the fundus are concerned, but that, when it is mainly attached to the fundus, particularly if the funis have been dragged upon, the description of Schultze is applicable to at least one of the modes of delivery. But this latter mode is often attended by considerable hemorrhage. Every one must admit that any facts which bear upon this stage of labor possess a highly practical interest.

In conclusion, let me mention that investigations have been carried out with a view to determine the size of the fetus, and also the sex of the fetus in utero. Ahfeld, of Leipsic, has attempted to ascertain the size of the child, in order to calculate the period of gestation, and more especially to make out before labour if any disproportion exists between the fetus and the genital passage. He pays special attention to the size of the head, the hardness of the bones which form it, and the condition of the sutures and fontanelles. The patient being placed on the back, with the thighs flexed, if the long axis of the uterus be from above down, the position of the fundus uteri is marked on the abdomen, and then one arm of Baudelocque's pelvimeter is guided by the finger along the vagina to the occiput of the child. The length from head to breech is thus ascertained, and by doubling this we have the entire length of the child. Ahfeld has collected the results of measurements made in two hundred and fifty cases, and gives much interesting information. In one of the tables which he gives, the relation between the length of the child and the two transverse diameters of the head is recorded. So far as these investigations go they are very valuable.

The interesting fact that the sex of the child can in a large proportion of cases be ascertained by auscultation during pregnancy has also lately been made known. When the fetal pulsations number 144 per minute, the child is probably a female, When they are 124 per minute, probably a male. It is said that a little variation from 124 upward, and from 144 downward, will not alter the diagnosis, provided auscultation be practised toward the end of pregnancy. Steinbach was correct in forty-five out of fifty-seven cases which he examined, while Frankenhäuser was right in all the fifty cases which he examined with a view to determine the sex of the child—*Obstetric Journal of Great Britain and Ireland.*

